# Development of a deep-learning phenotyping tool for analyzing image-based strawberry phenotypes

**DOI:** 10.3389/fpls.2024.1418383

**Published:** 2024-07-12

**Authors:** Jean Nepo Ndikumana, Unseok Lee, Ji Hye Yoo, Samuel Yeboah, Soo Hyun Park, Taek Sung Lee, Young Rog Yeoung, Hyoung Seok Kim

**Affiliations:** ^1^ Smart Farm Research Center, Korea Institute of Science and Technology (KIST), Gangneung, Republic of Korea; ^2^ Department of Plant Science, Gangneung-Wonju National University, Gangneung, Republic of Korea

**Keywords:** deep learning, strawberry, phenotyping, YOLOv4, U-net

## Abstract

**Introduction:**

In strawberry farming, phenotypic traits (such as crown diameter, petiole length, plant height, flower, leaf, and fruit size) measurement is essential as it serves as a decision-making tool for plant monitoring and management. To date, strawberry plant phenotyping has relied on traditional approaches. In this study, an image-based Strawberry Phenotyping Tool (SPT) was developed using two deep-learning (DL) architectures, namely “YOLOv4” and “U-net” integrated into a single system. We aimed to create the most suitable DL-based tool with enhanced robustness to facilitate digital strawberry plant phenotyping directly at the natural scene or indirectly using captured and stored images.

**Methods:**

Our SPT was developed primarily through two steps (subsequently called versions) using image data with different backgrounds captured with simple smartphone cameras. The two versions (V1 and V2) were developed using the same DL networks but differed by the amount of image data and annotation method used during their development. For V1, 7,116 images were annotated using the single-target non-labeling method, whereas for V2, 7,850 images were annotated using the multitarget labeling method.

**Results:**

The results of the held-out dataset revealed that the developed SPT facilitates strawberry phenotype measurements. By increasing the dataset size combined with multitarget labeling annotation, the detection accuracy of our system changed from 60.24% in V1 to 82.28% in V2. During the validation process, the system was evaluated using 70 images per phenotype and their corresponding actual values. The correlation coefficients and detection frequencies were higher for V2 than for V1, confirming the superiority of V2. Furthermore, an image-based regression model was developed to predict the fresh weight of strawberries based on the fruit size (R^2^ = 0.92).

**Discussion:**

The results demonstrate the efficiency of our system in recognizing the aforementioned six strawberry phenotypic traits regardless of the complex scenario of the environment of the strawberry plant. This tool could help farmers and researchers make accurate and efficient decisions related to strawberry plant management, possibly causing increased productivity and yield potential.

## 1 Introduction

The cultivated strawberry *Fragaria × ananassa* Duchesne is the most economically essential soft fruit worldwide, and its production and consumption are increasing in many parts of the world, including Korea (Simpson, 2018; Menzel, 2020). Given their significance in the global market and mounting year-round demand, strawberries are intensively grown under protected structures to ensure seasonal earliness, high-quality yield, and a continuous annual supply. Strawberry cultivation in Korea is primarily concentrated in greenhouses and is significant in the country’s agricultural industry and economy (Hwang et al., 2020). However, cultivating strawberries under such conditions requires extensive input and labor (Ilyas et al., 2021; Khammayom et al., 2022; Mbarushimana et al., 2022). To generate higher outputs and make tangible profits, farmers should optimize resource use efficiency through consistent and timely plant monitoring and make accurate farm management decisions.

Early identification and timely quantification of key plant phenotypes may provide valuable insights that can predict subsequent stages of plant development and critical outcomes such as yield. In the case of strawberry farming, the measurement of phenotypic traits, such as crown diameter (CD), petiole length (PL), plant height (PH), flower, leaf, and fruit size, is common among growers and researchers, serving as phenotypic markers employed to monitor plant growth balance and manage cultivation conditions. The crown size of strawberry seedlings during the transplanting stage has been established as a reliable indicator of post-transplantation vigor, and transplants with initially larger CDs are associated with high-yield strawberry components (Fridiaa et al., 2016; Fagherazzi et al., 2021). PL and PH are used to assess the overall growth potential of strawberries, and the petiole size can be used as an indicator of plant dormancy, where the plant produces shorter petioles in dormant conditions (Robert et al., 1999; Sønsteby and Heide, 2006). Similarly, PH is considered an index of plant management among strawberry producers (Takahashi et al., 2020). The leaf size is vital as, and in addition to photosynthesis and transpiration, it guides cultural practices, such as plant training, pruning, irrigation, and nutrition supply (Takahashi et al., 2020; Zheng et al., 2021). In addition, the analysis of leaf area and climatic variables can be used to predict plant evolution and the quality of strawberry fruits (de Castro et al., 2020). Similarly, it has been argued that increased leaf size and number may cause an increased fruit yield (Ahn et al., 2021). Flower and fruit size are significant factors in strawberry plant productivity and yield predictions (Chen et al., 2019; Menzel, 2020). To acquire growth information on the abovementioned phenotypic traits, most growers rely on traditional visual and manual phenotyping approaches, which are highly criticized for being subjective, destructive, and error-prone (He et al., 2017; Mahmud Sultan et al., 2020). Thus, to overcome these limitations, farmers of intensive cash crops, such as strawberries, and researchers need a robust, fast, and cost-effective phenotyping tool to facilitate their daily farm management based on quantitative phenotypic data during the plant’s life cycle.

Plant phenotyping combined with computer vision approaches provide better non-destructive options for plant monitoring through quantitative and qualitative analyses of complex plant traits, such as plant morphology, plant stress, crop yield, and plant physiological and anatomical traits (Costa et al., 2019). In the current state-of-the-arts in plant phenomics, many plant phenotyping methods are available, among which visible spectral imaging combined with deep-learning (DL) techniques present reliable advantages regarding affordability and quick measurement owing to the availability of various plant phenotyping hardware and software systems that facilitate image registration, processing, and data extraction (Fiorani and Schurr, 2013). Neural network-based DL techniques can be used to extract and analyze meaningful information on various plant traits from many collected image data; therefore, it is proposed that DL will dominate the future trends in image-based plant phenotyping (Tsaftaris et al., 2016). Among the recently published studies where DL techniques were applied for plant phenotyping, it is notable that convolutional neural networks, such as You Only Look Once (YOLO, also called single-shot detectors) and U-net, are among the most extensively used methods for object detection and segmentation (Zhang et al., 2018; Zheng et al., 2019; Ni et al., 2020).

Studies involving a combination of image processing techniques and computational intelligence to acquire strawberry growth phenotypic information in the field or laboratory settings using various sensors and platforms of different scales have been conducted focusing on detection (Gan et al., 2020; Zhou et al., 2020; Shin et al., 2021), segmentation (Pérez-Borrero et al., 2020; Perez-Borrero et al., 2021), classification (Feldmann et al., 2020; Aish, 2021; Fatehi and Akhijahani, 2021), and quantification (Lee et al., 2017; de Castro et al., 2020). However, most available phenotyping methods for strawberries are research-scale, costly, and unaffordable for ordinary profit-oriented farmers or researchers with limited financial means. Additionally, although DL techniques have been explored in strawberries, they are limited mostly to qualitative fruit attributes, and extensive studies embracing major strawberry growth and development indicators, such as CD, plant height, leaf size, and fruit size, remain unavailable. Therefore, developing a cheap, precise, and high-throughput phenotyping tool that covers more parameters is essential and should sustainably advance farming efforts in the strawberry sector.

In this study, we developed a Strawberry Phenotyping Tool (SPT) based on deep learning (DL), which integrates two prominent DL architectures notably “YOLOv4” and “U-net” into a single system. This image-based tool was designed for phenotyping and data analysis of strawberry plant phenotypic traits focusing on six important growth and yield traits: plant height, petiole length, crown diameter, leaf characteristics (area, length, and width), as well as flowers and fruits. The accuracy and reliability of this tool was enhanced by increasing the number of training images and diversified annotation techniques. We expect that if SPT is integrated into the current strawberry farming systems, it is likely to alleviate various farm management existing challenges and boost strawberry farmers’ productivity.

## 2 Materials and methods

### 2.1 Training image datasets acquisition

The strawberry-image-based SPT was developed using strawberry images collected from the Korean domestic cultivar ‘Seolhyang’, which was grown in the greenhouse facility of the Korea Institute of Science and Technology, Gangneung-si, Gangwon-do, Republic of Korea. Images of six phenotypes (crown, plant height, leaf, leaf petiole, flower, and fruit) of agronomic significance for strawberry growth and yield were acquired at variable distances using modern smartphones (iPhone 6S Plus, Apple Inc., United States and Galaxy S8 Samsung Electronics, South Korea) with iOS and Android operating systems for several days during the daytime (6:00–18:00) throughout the winter growing season between September 2019 and May 2020. The sampling devices used (smartphones) had both cameras of 12-megapixel, the exposure parameter was set automatically, and the objective focus system was set to autofocus mode.

In each instance, the image was acquired by steadily holding the Quick Response (QR) marker beside (parallel to) the target object, with the smartphone camera held perpendicularly against it, obtaining an image that included the target object and the QR marker. Spatial calibration for our measurement algorithm was conducted using a QR code (4.7 cm x 4.7 cm) as a reference. Both the growth parameters and the QR code were captured in a single image. The algorithm then recognized the QR code and its four corner points in the image, and applied distortion correction and length conversion based on those points for spatial calibration. The correct positioning and pose of the QR marker are critical for accurate image analysis (Teoh et al., 2022). Incorrectly positioned QR markers can cause overestimation or underestimation of the size of objectives captured in the same image (Data not shown).

Each phenotypic parameter has key areas detected or segmented by the deep learning model. Once these areas are detected, the distances between them are measured, and the lengths are converted based on the pixel size calibrated by the QR marker. Therefore, the parts that need to be parallel to the QR marker vary slightly for each phenotypic parameter. When the leaf area is calculated from the image, the entire leaf surface must be aligned and flat, parallel to the QR marker in the same image. These features also require hand support from the person taking the measurements during the process of acquiring phenotypic images. The SPT was designed for a single user to measure strawberry plants independently. Therefore, one hand holds the camera while the other hand holds the plant part parallel to the QR marker. This operation works smoothly when the strawberry plants are managed properly through the conventional pruning and defoliation. Our experiment was conducted under conventional strawberry cultivation practices where the SPT could operate smoothly. We provide explanations and example photos in Appendix A on how to position the QR marker for each phenotypic parameter during strawberry phenotyping, as well as how to perform the necessary hand support actions.

We initially collected 7,116 images to develop the first version of SPT (V1), and 7,850 images were used to construct the second version of SPT (V2). To obtain an RGB image dataset with a thorough variability of strawberry phenotypes under their natural habitat, the images were collected under different light intensity conditions (cloudy or sunny) and interferences, different days, and different growth stages. For each parameter, the shooting angle and shooting distance were continuously changed to collect images with various colors, postures, sizes, and backgrounds. The collected images were in the JPEG format, manually transferred, and stored in a computer for further processing. The detailed implications of each target phenotypic trait for strawberry farming and management are summarized in **Table 1**.

**Table 1:** Targeted strawberry phenotypes for imaging and features extracted.

Target phenotype	Features extracted in the image	Possible application of phenotyping results
1. Crown	Crown diameter (CD)	Strawberry yield is linked with the initial crown size (Torres-Quezada et al., 2015). Continuous monitoring of CD can provide early signs (prediction) of a plant’s vigor and yield.
2. Plant height	Height of the plant (PH)	Tracking strawberry growth strength and speed through PH monitoring (Takahashi et al., 2020).
3. Petiole	Length of the petiole (PL)	PL size is linked to plant activity status (Robert et al., 1997). Dynamic size changes in PL of the strawberry leaf can be used to determine the fate of strawberry plants under field conditions.
4. Leaf	-Leaf area (LA) -Leaf length (LL) -Leaf width (LW)	Leaf size attributes (LA, LL, and LW) can assist in the modeling of photosynthesis, evaporation, and evaluation of crop growth and productivity (Takahashi et al., 2020; Zheng et al., 2021; Jo et al., 2022).
5. Flower	Flower area (Fl. A)	Non-destructive prediction of strawberry qualitative and quantitative yield based on flower number and size (Chen et al., 2019).
6. Fruit	Fruit area (Fr. A)	Non-destructive prediction of strawberry qualitative and quantitative yield based on fruit number and fruit size (Hortyński et al., 1991).

### 2.2 Image dataset construction, annotation, and model training

**Figure 1:**
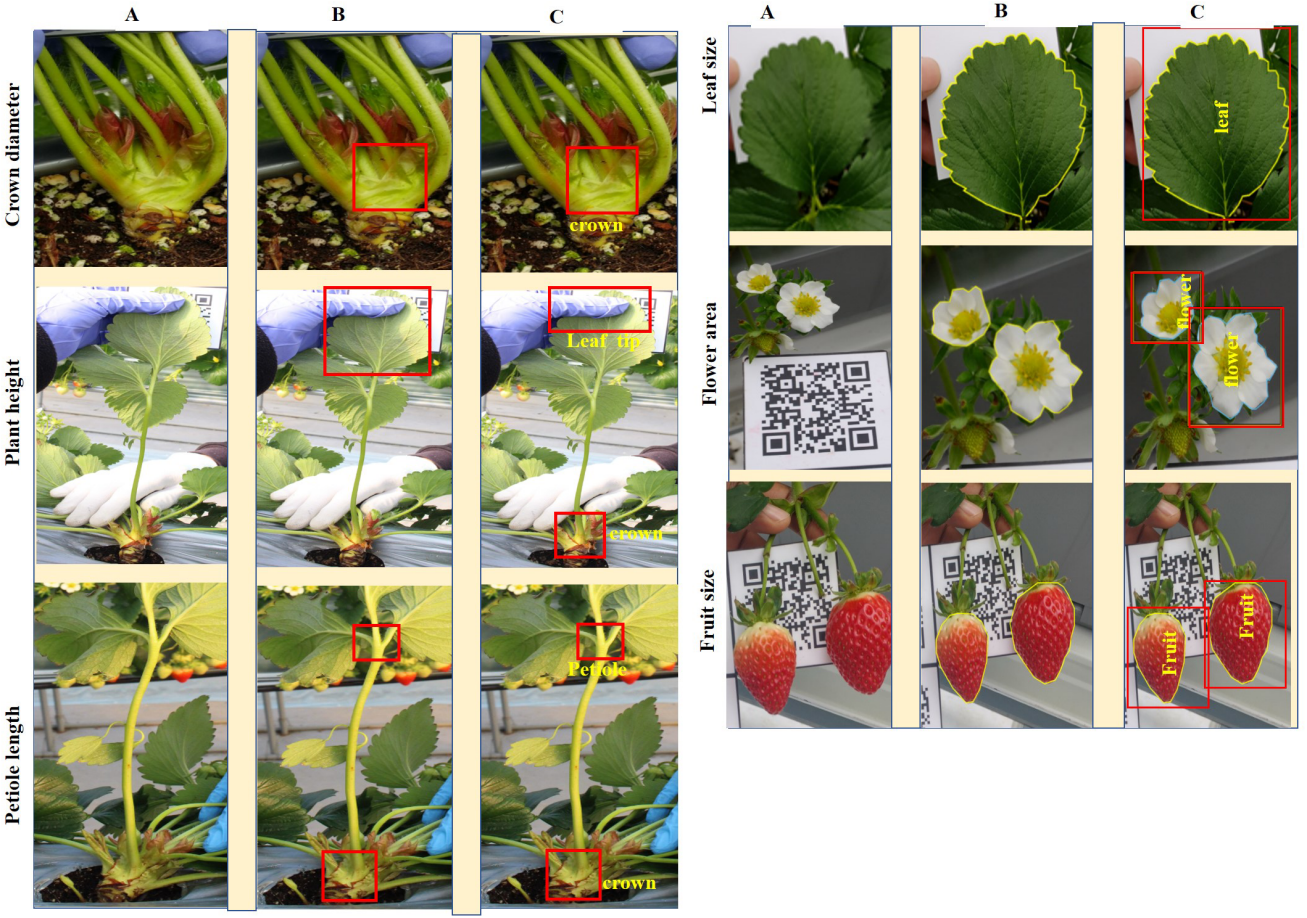
Annotation approaches for the six target phenotypic traits. **(A)** displays the raw images of six phenotypic traits: crown diameter, plant height, petiole length, leaf size, flower size, and fruit size. **(B)** shows the annotations for Version 1 (V1), where traits are marked with bounding boxes for crown diameter, plant height, and petiole length, and with polygons for leaf, flower, and fruit sizes. **(C)** illustrates the improved annotations for Version 2 (V2), which include descriptive labels for each trait and employ multi-target annotations within a single image when multiple traits are present.

**Figure 2:**
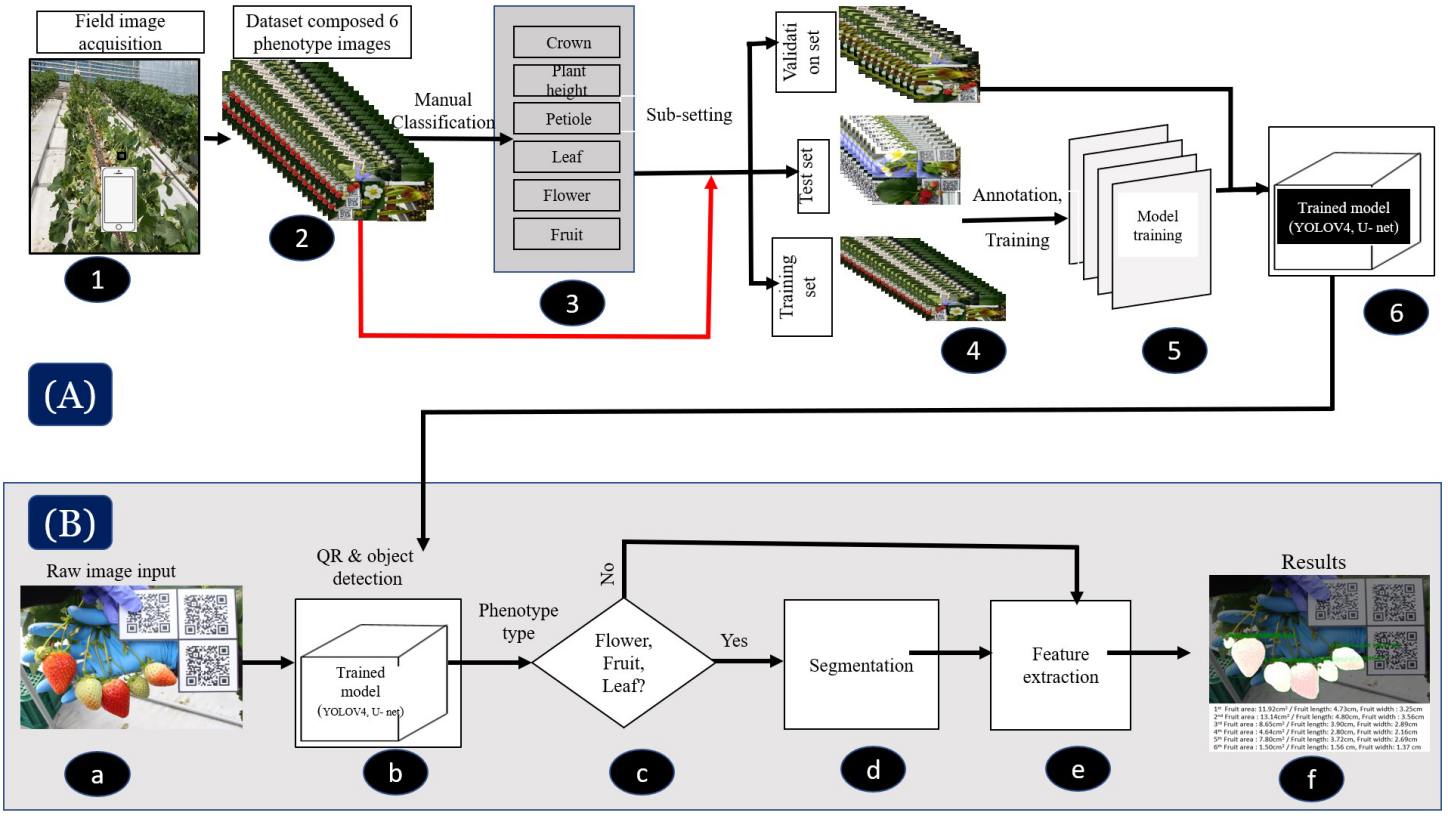
Flowchart of the deep learning-based phenotyping tool (SPT), which comprises two primary models: YOLOv4 and U-Net. **(A)** Illustration of the image acquisition, processing, and training procedures of the models for both versions. In Version 1, the models were trained through six processes (1–6), while in Version 2, they were trained through five processes (1, 2, 4–6). **(B)** (a–f) depicts the strawberry phenotyping analysis pipeline workflow using SPT, which involves image acquisition, detection, classification, segmentation and/or feature extraction, and results visualization.

Our SPT was developed primarily through two phases subsequently named versions (V1 and V2). The primary distinction between V1 and V2 lies in the volume of image data and the specific annotation method employed during their development. The number of images used to develop the two versions of the current phenotyping tool is presented in **Table 2** and the annotation principles adopted for each version are illustrated by **Figure 1**. A batch of 7,116 images was initially collected and manually classified according to their phenotypes before annotation. **Figure 2A** shows the workflow of the two versions of the model training process. The original dataset was divided into training, validation, and test datasets at 8:1:1 ratio. Subsequently, the test set was excluded from the training set. The VGG Image Annotator (VIA) tool (version 1.0.5), an image annotation tool developed by Dutta and Zisserman (2019), was used to manually annotate the objects of interest to obtain ground truth information for the subsequent training of V1. Because our system was built based on YOLOv4 (Bochkovskiy et al., 2020) and U-net (Ronneberger et al., 2015), an appropriate annotation technique that suffices for training these two architectures was considered. The YOLO algorithm series requires bounding box annotation for object localization to identify and detect specific objects in images. In contrast, U-net requires a class label and a pixel-level mask with an outline annotation of an object for semantic segmentation. Therefore, the annotation principle adopted to train these DL architectures needed to satisfy the above conditions. For phenotypic traits, such as crown, plant height, and petiole length, the regions of interest were annotated using a bounding box, whereas other phenotypes, such as leaves, flowers, and fruits, were annotated using polygon-shaped regions (**Figure 1**). To annotate the CD, a bounding box enclosing the thickest part of the strawberry crown was drawn with the lower side passing through the point of attachment of the leaves to the crown. To annotate the PH, two bounding boxes were used, with each placed at the bottom (crown) and the top (leaves) of the plant’s most extreme boundaries. The PL was annotated by drawing two bounding boxes, with each at the plant’s point of attachment of the leaves to the crown and at the point of attachment of the three leaflets to the petiole. The leaves, flowers, and fruits were annotated by carefully drawing polygons around the middle leaflets, flowers, and fruits, respectively. After annotation, the annotation information was downloaded and saved in CSV format and used to train and create the first version of our system.

**Table 2:** Number of images collected and annotated for each version of the Strawberry Phenotyping Tool (SPT).

Phenotypic trait	Number of images used for model development		Number of images used for validation	
	Version 1	Version 2	Version 1	Version 2
1. Crown	698	950	70	70
2. Plant height	804	940	70	70
3. Petiole	779	915	70	70
4. Leaf	1,490	1,560	70	70
5. Flower	853	923	70	70
6. Fruit	2,492	2,562	70	70
Total	7,116	7,850	420	420

Owing to the poor performance of V1, two approaches have been adopted to improve and change it to V2. First, we increased the number and variability of the training datasets. A total of 734 additional images were previously collected differently and added to the previous batch to make up 7,850 images. The PH and PL were targeted and acquired from a single image. Second, the annotation method was changed using an updated VIA version (VIA 2.0.10). Unlike V1, to annotate the second batch of images, all images were mixed to make a single folder, and all the objects to be annotated were pre-defined in the VIA 2.0.10 annotator and assigned class labels (**Figure 1**). Therefore, because more than one target objects co-occur in a single image, more than one target could be annotated in the same photograph (multitarget annotation). Similarly, to V1, the training, validation, and testing sets were fixed at 8:1:1 ratio. The resulting annotation information was downloaded, saved in JSON format, and used for V2 training and construction.

The images, annotation results and DL models subjected to V1 and V2 of STP are available at https://github.com/kist-smartfarm/SPT.

### 2.3 SPT SW architecture: YOLOv4 and U-net-based detection and segmentation pipeline and features extraction

The strawberry phenotype analysis pipeline workflow of the SPT is displayed in **Figure 2B**. We measured the phenotypic traits of strawberries in two ways using our system. First, the CD, PH, and PL were measured based on object detection (i.e., rectangular boxes). Second, the areas of flowers, fruits, and leaves were measured via object detection and segmentation (i.e., rectangular boxes and pixel-wise classification). As the U-net and YOLOv4 frameworks function differently, we designed a combination of YOLO v4 and U-net networks into a single system to build a robust and standalone SPT. YOLOv4 (Bochkovskiy et al., 2020) is a high-precision, real-time, one-stage object detection algorithm that involves using previous YOLO algorithms (Redmon et al., 2016; Redmon and Farhadi, 2017, 2018) with CSPDarknet53, PANet, and mosaic data augmentation. YOLOv4 performance was improved in previous YOLO algorithms through experiments and showed good performance in detecting small objects. Thus, we adopted this algorithm because it is suitable for detecting small objects, particularly strawberry fruits. For strawberry flower, fruit, and leaf measurements using our system, U-net-based semantic segmentation was adopted. U-net was designed to segment biomedical images in the original study (Ronneberger et al., 2015). The network is robust to small and thin objects, such as flowers, fruits, and petioles. Segmentation (i.e., pixel-wise classification) was performed using the object detection results, that is, a cropped detection image. Subsequently, we used our system to calculate the number of pixels (i.e., the area) based on the segmented results. Finally, all measurement results (e.g., length and area) were converted from pixels to actual distance or area using the detected QR code information. Our SPT equipped with the V2 DL was implemented on the web (https://www.cultigrowth.com) for the test of SPT with the independent datasets. The general information about how the SPT analyzes the studied strawberry phenotypic traits is found in section 3.5. For more detailed instructions on using the cloud-based SPT on the Cultilabs homepage, refer to the appendix A.

### 2.4 Ground truth data acquisition

To validate the functionality of our DL-based phenotyping tool, 70 plants (bearing flowers and fruits) were sampled from the Korean Institute of Science and Technology strawberry smart farm during the experimental period, and 70 images for each of the six phenotypes (crown, petiole, plant height, leaf, flower, and fruit) were captured using a smartphone camera and a QR marker. The resultant image files were renamed sample-wise to facilitate tracking and maintained for subsequent use as a validation dataset for the developed phenotyping tool. On the same day, after collecting digital images, the corresponding real data were acquired from the same plant parts directly through manual measurements or indirectly using phenobox-based methods (Czedik-Eysenberg et al., 2018). Manual data were collected using a measuring tape (PL and PH) and an electronic clipper (CD). The values of the remaining parameters, such as leaf area, leaf length, leaf width, flower area, and fruit area, were extracted from the phonebox-captured images using the Plant Analysis tool (Korea Scientific Technique Industry, Suwon, Republic of Korea). Additionally, the fresh weight of strawberries from the fruit cluster samples was recorded. The measured weight was used to construct a prediction model for strawberry fresh weight based on fruit size using our digital phenotyping tool. To evaluate the effectiveness of the Strawberry Phenotyping Tool (SPT) in monitoring and managing strawberry plants under greenhouse conditions, 'Seolhyang' strawberry cultivar transplants were sourced from a professional farmer in Pyeongchang, Gangwon-do, Republic of Korea. These transplants were planted at the Korean Institute of Science and Technology hydroponic greenhouse located in Gangneung-si, Gangwon-do, during the winter season from September 24, 2021, to April 30, 2022. The plants were cultivated in rectangular multi-potted containers measuring 20 cm x 15 cm x 60 cm, each equipped with six holes. The pots, filled with a soilless commercial medium and planted with ‘Seolhyang’ seedlings, were placed on 1-meter-high raised beds to enhance management accessibility. Cultivation was conducted using a standard hydroponic system, in compliance with established protocols for Korean strawberry farming within controlled environments. Weekly data on crown diameter, petiole length, plant height, and the sizes of leaves, flowers, and fruits were collected using both SPT and conventional tools on 27 randomly selected samples throughout the cultivation period.

### 2.5 Statistical analysis

After the completion of training, both models underwent consistent validation using a reserved subset of the dataset that had not been used during training. To evaluate the accuracy and reliability of the Strawberry Phenotyping Tool (SPT) throughout the development process, Pearson correlation coefficients and visualization techniques were utilized to compare measurements obtained from the SPT with those obtained conventionally. Paired *t*-tests were then employed to statistically validate the improvements achieved by different versions of the SPT, determining which version produced values most comparable to those obtained conventionally. These tests were crucial for assessing whether the enhancements in phenotypic detection by each version of the SPT were significantly different from those obtained through conventional measurements.

During the field validation stage, the impact of phenotypic variations on fruit yield was explored by categorizing data collected from various phenotypic traits into distinct size clusters before fruit harvesting. For example, at the transplantation stage, crown diameter (CD) was categorized into three clusters: Cluster 1 for samples with large crowns, Cluster 2 for samples with medium crowns, and Cluster 3 for samples with small crowns. The K-means clustering algorithm was utilized to ensure effective categorization based on phenotypic sizes. Similarly, critical phenotypic parameters such as total flower area per plant, total weight of unripe fruits per plant, and leaf area per plant were also clustered. This clustering facilitated structured comparisons of weekly yields across different phenotypic categories. The comparisons were conducted using analysis of variance (ANOVA) and Tukey’s Honestly Significant Difference (HSD) test to determine the statistical significance among the groups at a significance level of *p*<0.05. These comparisons allowed us to explore whether larger phenotypic sizes correlated with higher yields.

## 3 Results

### 3.1 Dataset size and annotation method effect on DL performance

**Figure 3:**
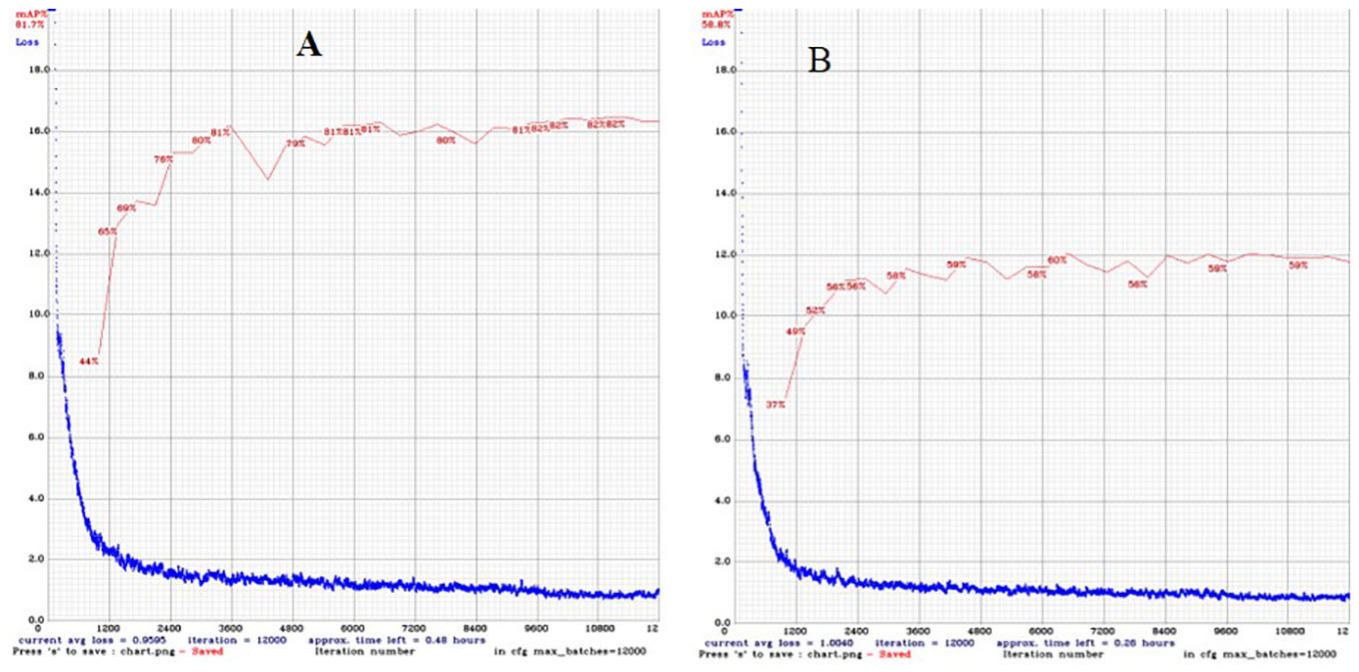
Change in training loss and validation mean precision average with the number of epochs of **(A)** Version 1 and **(B)** Version 2 using the training dataset.

**Figure 4:**
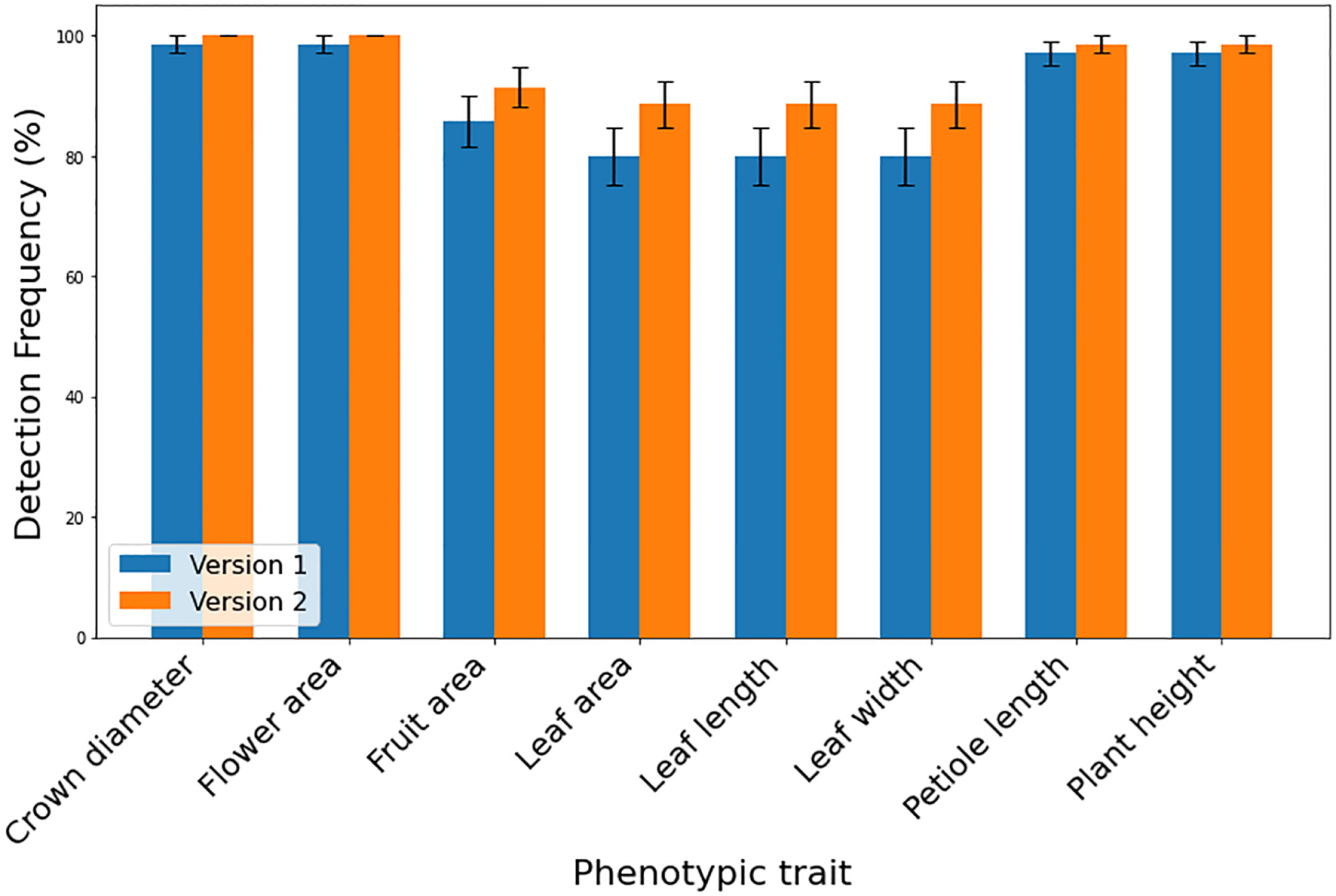
Detection frequencies of various strawberry target phenotypic traits using different SPT versions (V1 and V2) (n=70) displayed as percentages. Error bars shows the standard error of the detection frequencies.

The DL-based strawberry phenotyping digital tool was developed in two primary steps (subsequently called versions). The difference between these two versions is primarily owing to the different numbers of images and annotation techniques used during the development process. **Table 2** presents the different numbers of images collected per phenotypic trait and used to develop the two SPT versions, and **Figure 1** shows the annotation principle adopted correspondingly. V1 was initially developed using 7,116 images representing various phenotypic traits of interest. Annotation was performed by drawing a bounding box encompassing the target part for the CD, PL, and PH, and a polygon was drawn to include leaf, flower, and fruit areas. Because of the low detection frequency and precision of SPT-V1, we increased the number of images to 7,850 and changed the annotation techniques which led to V2. In V1, the bounding boxes for annotating the upper boundaries for PH and PL were drawn such that all leaves were enclosed inside the boxes; in V2, these boxes were reduced to include only the highest leaf part for PH and the junction point of the petiole and its leaflets for PL. Additionally, while in V1, each phenotype was annotated individually for each image without labels, in V2, more than one phenotype was annotated in one image. The resulting V2 of SPT showed higher detection precision (**Figure 3**) and frequency (**Figure 4**).

### 3.2 Ground truth versus image-based plant phenotype measurements

The measurements obtained through conventional methods (measured values) were systematically compared with those predicted using the two versions of the Strawberry Phenotyping Tool (SPT) (predicted values). Two analytical approaches were utilized to ensure robust evaluation and avoid potentially misleading conclusions based on the data presented in **Table 3**.

**Table 3:** Comparison of strawberry phenotypic traits measured conventionally and with SPT-V1 and SPT-V2.

Phenotypic trait	Measurements of phenotypic trait			P-values	
	Conventional	SPT-V1	SPT-V2	Conventional vs. SPT-V1	Conventional vs. SPT2
1. Crown diameter (mm)	14.36 ± 2.62	14.84 ± 2.56	15.23 ± 2.62	0.027*	0.058
2. Plant height (cm)	24.62 ± 5.36	22.95 ± 5.01	23.17 ± 5.09	0.038*	0.051
3. Petiole length (cm)	15.09 ± 4.49	14.61 ± 4.06	14.87 ± 4.10	0.192	0.322
4.1. Leaf area (cm^2^)	25.67 ± 8.65	25.9 ± 7.26	27.10 ± 9.49	0.437	0.187
4.2. Leaf length (cm)	7.04 ± 1.29	6.39 ± 1.08	6.56 ± 1.18	0.001*	0.015*
4.3. Leaf width (cm)	5.51 ± 1.02	5.11 ± 0.77	5.31 ± 0.94	0.007*	0.17
5.1. Flower area (cm^2^)	3.26 ± 1.04	3.05 ± 1.08	3.06 ± 1.04	0.287	0.311
6. Fruit area (cm^2^)	6.52 ± 3.73	6.32 ± 3.68	6.41 ± 3.72	0.311	0.372

Means ± standard deviation (SD) are shown for each variable, along with corresponding P-values for paired *t*-tests for comparing conventional measurements with the two SPT versions (SPT-V1 and SPT-V2) measurements. The p-values are presented in the last two columns. A p-value less than 0.05 (*) indicates a statistically significant difference between the two means.

**Figure 5:**
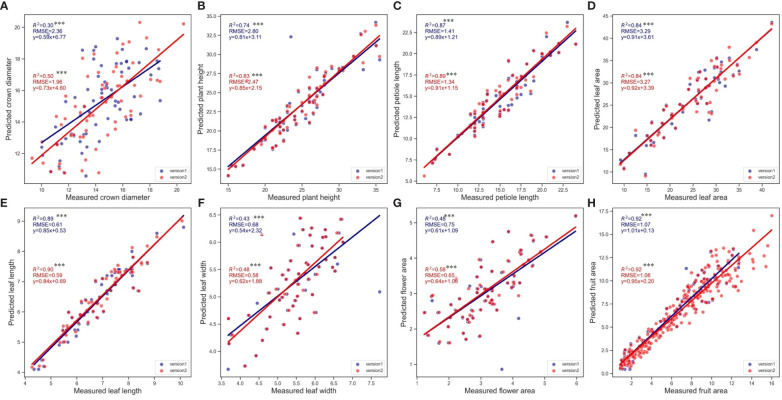
Correlation between real and predicted values (using V1 and V2 of SPT) for the six phenotypic traits. **(A)** Crown diameter, **(B)** Plant height, **(C)** Petiole length, **(D)** Leaf area, **(E)** Leaf length, **(F)** Leaf width, **(G)** Flower area, **(H)** Fruit area. Except for fruit where n=312. For the rest of the variables, n=70. Asterisks denote statistical significance at *p=0.05* (*** *p<0.001*).

Initially, the analysis was conducted using Pearson correlation analysis (**Figure 5**), which confirmed the positive correlation between the measured and predicted values for all examined phenotypic traits. The analysis revealed that the fruit area exhibited the highest linear correlation, demonstrating exceptional precision in predictions with the highest R² and relatively low RMSE values, emphasizing the tool’s accuracy in capturing this trait’s variability. On the other hand, crown diameter displayed the lowest linear correlations in both SPT versions. Highly statistical significance was confirmed for the correlations across all variables (*p<*0.001), reinforcing the reliability of the predictions made by the Strawberry Phenotyping Tool (SPT). Comparative analysis showed that Version 2 (V2) consistently demonstrated higher correlation coefficients (R²) and generally lower RMSE values than Version 1 (V1) across most phenotypic traits. This improvement highlights V2’s enhanced algorithmic performance and overall efficacy in predicting phenotypic traits more accurately. The results collectively underline the significant advancements in V2, offering more reliable and precise measurements critical for strategic strawberry plant monitoring and management under controlled farming systems

Furthermore, measured values were compared against the predictions made by SPT, focusing on the average values calculated for each phenotypic trait. Similarly, across both versions of the SPT, the predicted averages generally approximated the conventionally measured values closely. Specifically, except for leaf length, the average values predicted by SPT Version 2 did not significantly differ from the real values, as shown by *p*-values greater than 0.05. However, in SPT V1, notable differences were observed in traits such as crown diameter, plant height, leaf length, and leaf width where the average values differed significantly from the ground truth values, as indicated by *p*-values less than 0.05. This differential accuracy highlights the variations in the effectiveness of the two SPT versions when estimating specific phenotypic traits.

### 3.3 DL-based regression model for predicting strawberry fresh weight

**Figure 6:**
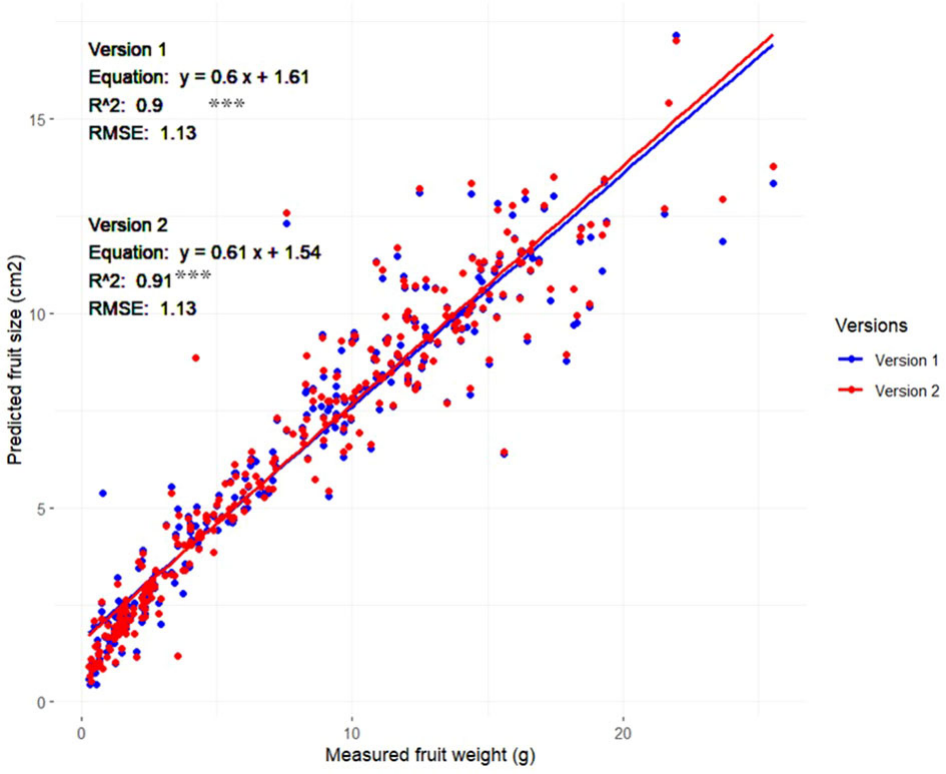
Relationship between strawberry fruit size (area) measured using SPT (Versions 1 and 2) and fruit weight (n=310) Asterisks denote statistical significance at *p=0.05* (*** *p<0.001*).

As previously detailed in the methodology section, our study employed a specialized dataset comprising 420 images, distributed evenly among the six target phenotypic traits, with 70 images dedicated to each trait. This dataset was intentionally prepared to evaluate the performance of the Strawberry Phenotyping Tool (SPT) across different developmental phases. Specifically targeting the ‘fruit size’ trait, strawberries from the samples corresponding to the fruit size image batch were harvested immediately after imaging to ensure data accuracy and freshness and, subsequently, measurement of each strawberry’s fresh weight was conducted using an HR-200 electronic balance (A&D Company, Limited, Tokyo, Japan) by ensuring that each fruit’s weight and its precise position within the images were carefully maintained. These fruits included fruits of various sizes and developmental stages and they were used to develop a regression model to predict strawberry fruit fresh weight based on the measured fruit area. The results are illustrated in **Figure 6** and show a strong positive correlation between the predicted fruit sizes from SPT and the actual measured weights. Both Version 1 and Version 2 demonstrated relatively similar results, with Version 1 achieving an *R* ^2^ value of 0.90 while Version 2 showed a slight improvement with an *R* ^2^ value of 0.91

### 3.4 SPT can be used to monitor strawberry growth and predict yield under greenhouse settings

**Figure 7:**
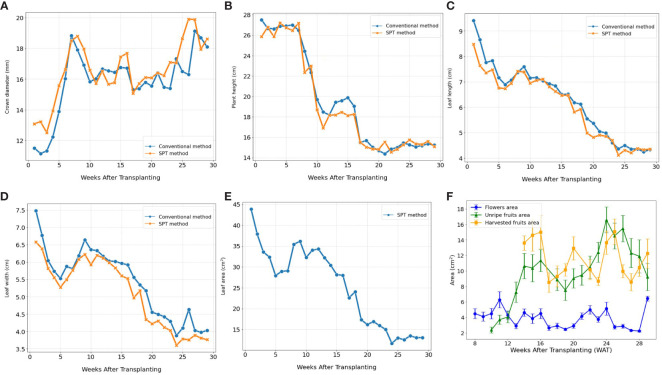
Illustrates the temporal dynamics of various phenotypic traits related to growth and yield in strawberry plants over the growing season. The phenotypic data plotted include, **(A)** crown diameter, **(B)** plant height, **(C)** leaf length, and **(D)** leaf width collected by both conventional and SPT methods. Additionally, **(E, F)** depict the evolution of leaf area, flower size, the area of unripe (non-harvestable) and ripe (harvestable) fruits using data exclusively collected by SPT. (n=27). Error bars represent the standard error of the mean.

The Strawberry Phenotyping Tool (SPT) was successfully utilized at the Korean Institute of Science and Technology hydroponic greenhouse to monitor the ‘Seolhyang’ strawberry cultivar. The tool proved to be as effective as traditional methods in tracking the growth and yield phenotypic parameters across 27 samples from September 24, 2021, to April 30, 2022. **Figures 7A–D** depicts how SPT accurately captured the temporal dynamics and patterns of crown diameter, plant height, leaf length, and leaf width, while also providing additional insights beyond the capabilities of conventional methods. Notably, SPT excelled in measuring leaf area and monitoring the occurrence and sizes of flowers and fruits. **Figure 7E** illustrates SPT’s ability to track the decrease in leaf area as plants matured, offering valuable insights into the leaf-changing pattern during the crop cycle. Furthermore, **Figure 7F** demonstrates SPT’s unique capability to monitor the sizes of flowers, unripe (non-harvestable), and ripe (harvestable) fruits over time, a feature unavailable with manual measurement methods. These advanced functionalities highlight the comprehensive understanding of phenotypic changes provided by SPT, crucial for effective crop management.

**Figure 8:**
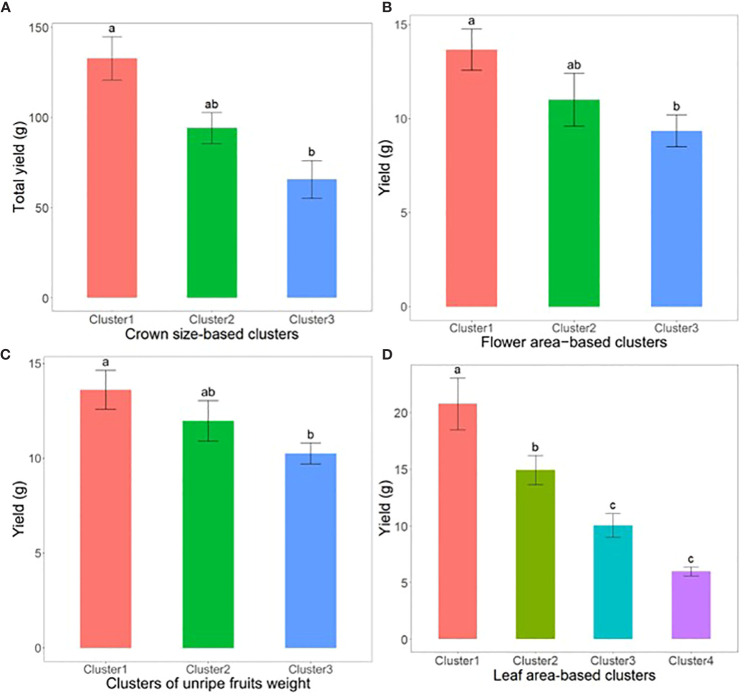
Illustrates the yield of strawberry fruits derived from plant samples sorted into phenotypic clusters based on size at critical growth stages. **(A)** showcases total yield per plant among crown size-based clusters, measured 2 weeks post-transplanting (n=22). **(B)** displays weekly yield per plant across flower size-based clusters, taken 5 weeks prior to harvest (n=125). **(C)** reveals weekly yield per plant for clusters categorized by the weight of unripe fruits, recorded 2 weeks before harvest (n=198). Lastly, **(D)** illustrates weekly yield per plant for leaf area-based clusters, assessed 4 weeks pre-harvest (n=80). The error bars represent the standard error, and groups labeled with the same letters within the same subfigure are not statistically different according to Tukey’s Honestly Significant Difference test, *p<0.05*.

Subsequently, we conducted an analysis using Analysis of Variance (ANOVA) and Tukey’s Honestly Significant Difference (HSD) test to explore the impact of early phenotypic size variation on strawberry fruit yield. For example, data segmentation based on crown diameter (CD) at the transplantation stage, categorized as large, medium, and small, showed that early crown sizes were statistically significant predictors of yield outcomes (**Figure 8A**). Additionally, when samples were categorized based on other important phenotypic parameters such as flower area, unripe fruits, and leaf area into different size-based clusters at specified times before fruit harvesting, a notable trend emerged: an increase in the size of these phenotypes was associated with a substantial increase in yield (*p*<0.05) (**Figures 8B–D**).

### 3.5 Data analysis and visualization using SPT

**Figure 9:**
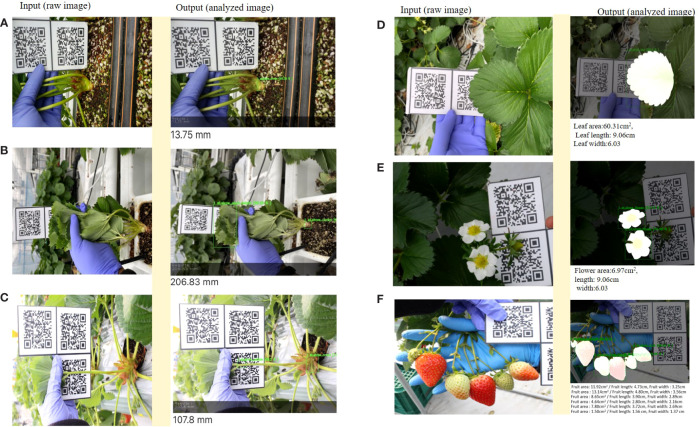
Example of data analysis and visualization (screenshots) for the six strawberry phenotypic traits (**A**. Crown diameter, **B**. Plant height, **C**. Petiole length, **D**. Leaf size, **E**. Flower size, **F**. Fruit size) using SPT. The left images (under input column) are the raw input images, and the right images (under output column) show the output of the analyzed images, with quantitative results at the bottom.

In this section, we describe the details related to the functionality of the SPT focusing on image data analysis and results visualization. Our phenotyping tool is used to analyze strawberry images and provides quantitative data in two alternative ways, and both require registration of the user as well the plant samples before usage. In addition, both methods require internet access. The one is a direct real-time method and operates on mobile phones. To acquire phenotypic data using this method, the user needs a smartphone and a QR code. Then chooses the plant ID and take the image of the target phenotype so that both the object and the QR code appear in the same image and confirm the task by clicking ok. The detected and analyzed phenotype is displayed along with the results within a short time (**Figure 9**). The second method is an indirect and works on both smartphone and computer. To analyze an image using this method, you choose the plant ID and the corresponding image to be analyzed is uploaded to the system from the computer or smartphone storage. The image should contain the target phenotype and the QR code. Once the image enters the system, the target objects are immediately detected and analyzed, and the results are displayed, in a manner similar to that of direct methods. The results can be downloaded as CSV files for further processing.

## 4 Discussion

In this study, we propose an image-based digital phenotyping tool, utilizing deep learning (DL), for analyzing strawberry phenotypes focusing on six essential parameters for strawberry growth and yield analysis. These images can be acquired using modern smartphones and analyzed directly at the field level or stored for subsequent analysis. Several machine-learning-based solutions for strawberry plant phenotyping to acquire growth and yield information have been proposed, most of which have concentrated on fruit detection, classification, segmentation (Chuah, 2018; Kirk et al., 2020; Basak et al., 2022; Wu et al., 2022), fruit size (Lee et al., 2017; Yue et al., 2020), and leaf area (de Castro et al., 2020). However, a comprehensive digital phenotyping tool to measure most agronomic traits of strawberry growth, such as CD, PH, and PL, remains unreported in the existing literatures. Therefore, the efficiency of this tool in the non-destructive extraction of strawberry growth and yield phenotypic data, including those that were not attempted digitally, makes it a promising tool that can assist farmers and ordinary researchers in proper decision-making. The core robustness of our system was rendered by integrating two DL architectures, YOLOv4 and U-net, which are reportedly efficient regarding high-precision and real-time object detection (Bochkovskiy et al., 2020) and image segmentation (Ronneberger et al., 2015), respectively. The combination of YOLO and U-net series together or with other architectures to build a more robust phenotyping system or for comparison purposes has also been successfully performed in other crops such as grape (Barbole and Jadhav, 2021), wheat (Ullah et al., 2021), and mango (Koirala et al., 2019).

During the development, the real data values of the six phenotypes were acquired using conventional approaches, and the corresponding images were maintained. The latter served as the benchmark dataset for evaluating the performance of our digital system throughout the development process. Upon completion of V1, most of the performance metrics assessed, such as the mean precision average, detection frequency and Pearson correlation coefficients, were relatively poor in V1. However, by increasing the number of training datasets and changing the annotation method from single-target no-labeling annotation to multi-target labeling annotation, the abovementioned metrics improved for most of the various phenotypic parameters studied, making up V2. Such improvements in the performance and robustness of our DL framework in V2 can be attributed to the consistency and multi-target labeling annotations. During data collection, several objects, including nontarget objects, may be included in the image. In such cases, if the nontarget object is similar to the target, it confuses the annotator. Therefore, the annotation criteria should be pre-defined and consistently maintained throughout the annotation process.

For example, if the target strawberry fruit and the fruit next (not the target) to it are of the same size or distance (distance from the camera), both fruits need to be annotated; however, if it is blurred and not clearly visible or it is distant from the camera, only the target fruit should be annotated. By creating such consistent criteria to avoid annotating far or invisible targets, it is possible to prevent the model from being incorrectly trained on objects that look almost similar when learning. In addition, in a single image, the co-occurrence of more than one but different objects is normal. For example, while capturing a fruit cluster, flowers may be captured together, or petioles may be included while capturing plant height.

By labeling and annotating all the available objects in the image (those investigated) and training the AI accordingly, the overall performance and robustness of the system were increased. Low-quality annotation severely affects training models (Guo et al., 2020). According to Mullen et al. (2019), the annotation technique impacts the deep neural network, and the inconsistency of the annotation technique may cause incorrect conclusions regarding model performance. Recently, Yun et al. (2021) applied multiple labeling annotations on ImageNet (Russakovsky et al., 2015), and without modifying the models, they improved the classification accuracy solely by revising the models from single-object labeling annotations to multi-labeling, which is consistent with our attempt to improve our phenotyping tool from V1 to V2. These findings are consistent with those reported in other studies (Barbedo, 2018; Shorten and Khoshgoftaar, 2019; Alhazmi et al., 2021) that dataset size significantly affects the performance of DL models. Furthermore, the strong correlation between strawberry fruit weight and fruit area (R^2^>0.9) confirmed the efficiency of our system for predicting strawberry fruit yield based on the measured fruit area. Such options will help users forecast yields non-destructively and accurately, which, in the case of professional farmers, can assist in planning harvesting and marketing activities. This model can also be applied to strawberry studies.

In this study, we evaluated the efficiency of the SPT in collecting strawberry growth and yield data under real field conditions. The results revealed that SPT was comparable to conventional approaches in collecting growth information and could be used to monitor phenotypic traits, such as leaf area, flower area, and fruit area, which were previously challenging to obtain using traditional methods. A relatively large increase in the size of the aforementioned phenotypes before harvesting was associated with a significant increase in the yield. These results underline the potential of the SPT as a valuable tool for farmers engaged in professional and smart farming and its significance in yield prediction. Accurate yield prediction is crucial for farmers, as Kerfs et al. (2017) reported that weekly strawberry yields can vary significantly and emphasized that farmers should regularly monitor their fields for smooth planning of farm operations, particularly postharvest activities, for adequate resource distribution. These findings are consistent with those of previous studies. For example, Abd-Elrahman et al. (2021) investigated the integration of ground-based canopy images into modeling approaches to improve strawberry yield. Using canopy images captured with a handheld digital camera and machine-learning algorithms caused a significant improvement in yield prediction accuracy compared with traditional approaches. Yoon et al. (2023) combined immature fruit information with AI techniques to develop a strawberry yield prediction model. They also concluded that DL-based strawberry fruit detection results could contribute to yield prediction. In a previous study (Hassan et al., 2018), hyperspectral remote sensing imagery was used to acquire leaf area index parameters and six other vegetation indices to investigate the relationship between these parameters and yield under different growing conditions, which revealed that the leaf area index was highly related to yield. These findings provide valuable insights into the relationship between leaf indices and yield and contribute to a better understanding of the factors influencing fruit productivity. Finally, using the data collected through the SPT, we investigated the impact of initial crown size on strawberry yield. Our analysis involved comparing the total yields obtained from different crown classes, including small, medium, and large crowns. Our results indicated that strawberries with a larger initial CD produced significantly higher yields than those with smaller crowns, confirming the findings of previous studies (Torres-Quezada et al., 2015; Fridiaa et al., 2016; Fagherazzi et al., 2021). These studies also reported similar results, suggesting that the initial crown size is a factor that significantly affects strawberry yield. Therefore, if SPT is integrated into strawberry farming, it will possibly alleviate various farm management-related challenges and boost farm production.

Although the increase of the dataset and enhancement of annotation techniques significantly improved the core models (YOLOv4 and U-net) of our Strawberry Phenotyping Tool (SPT) in terms of precise detection, measurement, and analysis of strawberry phenotypic features, we encountered several challenges that require improvements in future studies to maximize the full potential of the SPT.

The two core models of our Strawberry Phenotyping Tool (SPT), YOLOv4 and U-net, require a relatively large volume of data, which necessitates extensive labor to annotate such a dataset. Additionally, managing this large volume of image data and the related logistics remains a challenge due to significant computational resource requirements.

The current version of the SPT was unable to accurately detect petiole length under field conditions, resulting in the omission of these results from our report. Additionally, detecting and measuring relatively small features poses a significant challenge, often requiring multiple captures of the same object from different angles to ensure target feature detection and accuracy. This issue is more aggravated when the plant canopy becomes bushy in later growth stages, especially if excessive leaves and side crowns are not pruned and managed properly. If the plants overgrow too much, their size may also exceed the capacity of a single person to acquire the images effectively, especially for plant height Enhancing the SPT’s performance to provide more precise analysis of very small objects and operates well even in complex environments and growth conditions is a crucial area for future improvement.

Additionally, the SPT does not currently support the automatic categorization of fruits into different developmental stages, such as distinguishing between ripe and unripe fruits. Addressing this limitation could significantly enhance the tool’s utility and applicability.

Lastly, flower initiation is a critically important event for the successful cultivation and production of june-bearing strawberry plants, directly impacting fruit yield (Van Delm et al., 2014; Li et al., 2021). This transient phenomenon is challenging to predict and is traditionally confirmed through destructive sampling and microscopic observation. By training our SPT models on potential phenotypic traits speculated to be indicators of flower initiation at the nursery stage, and supplementing this with robust statistical analysis, we anticipate that the SPT will provide valuable insights into the key phenotypic traits indicative of vegetative-to-reproductive changes.

We acknowledge that YOLOv8 has been released since the completion of our study. YOLOv8 has been reported to achieve high accuracy and fast inference speed (Liu et al., 2024). However, we chose to use YOLOv4 in our work due to its well-established performance and balance for server compatibility in our application. YOLOv4 had been extensively tested and was well-documented for deployment on servers. This was crucial for our study as we had already established a YOLOv4-based server infrastructure for our smartphone-based web application.

While YOLOv8 may offer potential performance gains, YOLOv4 was a suitable choice considering these factors. We provide the complete raw datasets (images subjected to YOLOv4), along with the results of annotation and deep learning at https://github.com/kist-smartfarm/SPT, so that we and other research groups can use these datasets with the advanced deep learning architectures to develop more advanced practical phenotyping methods in future study.

Conclusively, in this study, a DL-based phenotyping tool was developed to collect, process, and analyze image-based strawberry phenotypes of six essential agronomic traits (CD, PL, PH, flower, leaf, and fruit size). The proposed approach involves integrating the YOLOv4 and U-net architectures into one system to make it more robust for better feature detection and extraction. An increased dataset size with various backgrounds, coupled with multi-labeling object annotation, improved the efficiency of our system in measuring target phenotypic traits with greater precision and accuracy. The evaluation of our phenotyping tool under real field settings showed the same efficiency in collecting strawberry growth data as conventional approaches, with additional capacity for predicting yield based on leaf, flower, and fruit indices. Real-time strawberry phenotyping with the current digitalized solution has potential applications in strawberry smart farming, assisting researchers and farmers in making appropriately informed decisions. In future studies, more phenotypic features, such as strawberry fruit maturity stage and canopy area quantification, should be added to the system to enable more in-depth strawberry phenotyping.

## Data availability statement

The datasets presented in this study can be found in online repositories. The names of the repository/repositories and accession number(s) can be found in the article/supplementary material.

## Author contributions

JN: Data curation, Formal analysis, Validation, Visualization, Writing – original draft, Writing – review & editing. UL: Conceptualization, Formal analysis, Methodology, Software, Validation, Writing – review & editing. JY: Data curation, Investigation, Visualization, Writing – review & editing. SY: Data curation, Formal analysis, Writing – review & editing. SP: Formal analysis, Methodology, Software, Writing – original draft. TL: Formal analysis, Software, Supervision, Visualization, Writing – original draft. YY: Conceptualization, Investigation, Methodology, Supervision, Writing – review & editing. HK: Conceptualization, Funding acquisition, Investigation, Project administration, Supervision, Writing – review & editing.
