# Supplementary material for: Development of a deep-learning phenotyping tool for analyzing image-based strawberry phenotypes
Source: Front Plant Sci. 2024 Jul 12;15:1418383. doi: 10.3389/fpls.2024.1418383 (PMC11284602; doi:10.3389/fpls.2024.1418383)
Supplement: Supplementary file 1 [file DataSheet_1.docx]

**Appendix A**

**User Manual for Cultilabs website-based Strawberry Phenotyping Tool (SPT)**

1. **Introduction**

This manual is a guide to using the Cultilabs website Strawberry Phenotyping Tool (SPT). Designed to empower users, this interface allows independent testing of strawberry datasets and conducting phenotyping activities to measure various plant traits. It offers a convenient and efficient way to analyze strawberry plant characteristics.

1. **System Requirements:**

To utilize the SPT for real-time (direct) phenotyping, ensure your device has a camera and QR code. For indirect phenotyping, any computer or mobile device with internet access suffices.

1. **Accessing Cultigrowth:**

To access the SPT, navigate to the Cultigrowth website at https://www.cultigrowth.com. If necessary, select "ENG" for the English language. Log in using the provided credentials:

Username: cultilabs3@test.com

Password: 1234

1. **Choosing the Project:**

Once logged in, select the project named "SPT_try," specifically designed for testing the strawberry phenotyping tool.

1. **Using the SPT:**

Depending on your needs, there are two options available:

**Option** 1: Real-time Strawberry Phenotyping:

This option requires a smartphone, tablet, or iPad with a camera. Simply select the plant ID, capture an image of the desired trait, ensuring both the QR code and target phenotype are visible, and upload the image. After a brief analysis, the results will be returned along with the image.

O**ption 2**: Indirect Method:

Similar to the real-time method, users upload pre-captured images containing the target phenotypic trait and QR code. These images should be stored on a computer, smartphone, or tablet device.

1. **How to take photos with a QR marker**

**General principle**

Generally, for accurately and precisely measuring the target strawberry phenotyping trait using SPT, it is crucial to position the camera (smartphone camera, etc.) so it directly faces the target at the same height. Place the QR marker next to the target, ensuring both are on the same plane and at the same distance from the camera. The QR marker should be aligned parallel to the target to avoid angular discrepancies. Capture the image so that both the target and the QR marker are clearly visible in the frame. Incorrectly positioned QR markers can lead to significant measurement errors, as shown in the accompanying figure. This procedure helps maintain consistency and accuracy in the measurements.

| Incorrect Methods | Correct Method |
| --- | --- |
| 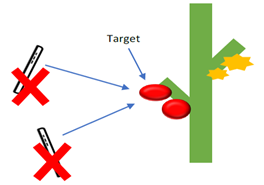 | 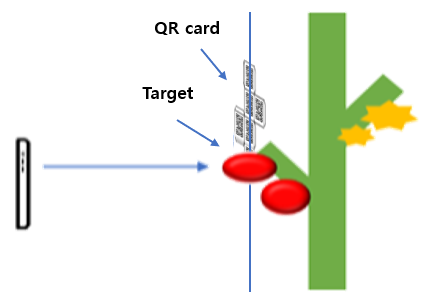 |

1. **Methodology for acquiring images for each target phenotypic trait**
2. **Plant height**

| **Phenotypic parameter** | **Measurement method** |
| --- | --- |
| 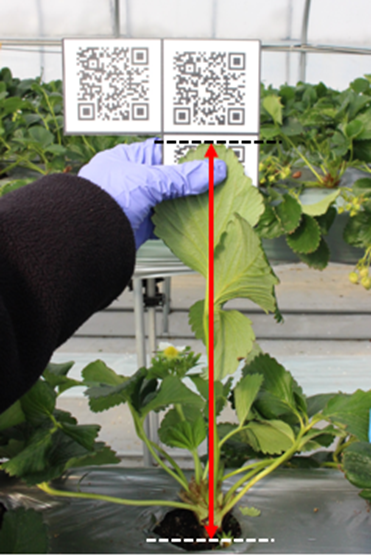 | To measure plant height, securely hold the QR marker with one hand and position it vertically and directly above the target plant, ensuring alignment with the plant's height. Pinch the plant's fully developed leaves or the tallest leaf with one hand at an adequate distance. The top edge of the leaf and the bottom of the branch (indicated by black and white dotted lines, respectively) should be clearly visible in the frame. using the other hand, capture an image that includes the entire plant, from the soil level to the tip of the highest leaf, as indicated in the provided illustration image. |

1. **Leaf (leaf area, length and width)**

| **Phenotypic parameter** | **Measurement method** |
| --- | --- |
| **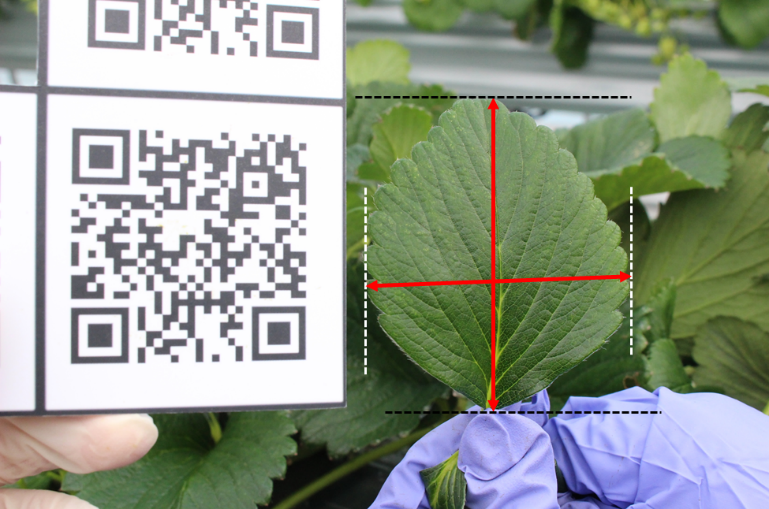** | To precisely measure the leaf size, hold the QR marker steadily with one hand, positioning it horizontally (also vertically, or obliquely based on the leaf's orientation). Ensure the marker is placed on top (or side) of the target leaf. Capture the image so that the entire middle leaflet is visible and not covered by the hand. Both the leaf length edge (black dotted line) and leaf width edges (white dotted lines) should be clearly visible in the camera, as indicated in the image. |

1. **Petiole length**

| **Phenotypic parameter** | **Measurement method** |
| --- | --- |
| 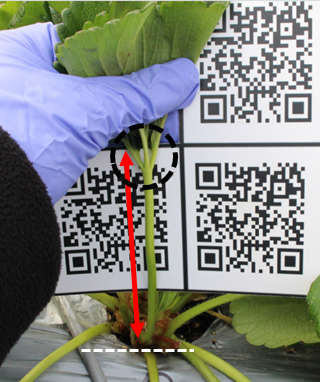 | To precisely measure the petiole length using a QR code marker, hold the QR marker steadily on the side of the target petiole, and take an image so that the petiole base (white dotted line) and the part where the leaflet petiolules start (black dotted circle) are visible, as indicated in the image. Ensure that both the QR code and these reference points are clearly visible within the camera frame to achieve precise calibration and accurate measurements. |

1. **Crown diameter**

| **Phenotypic parameter** | **Measurement method** |
| --- | --- |
| **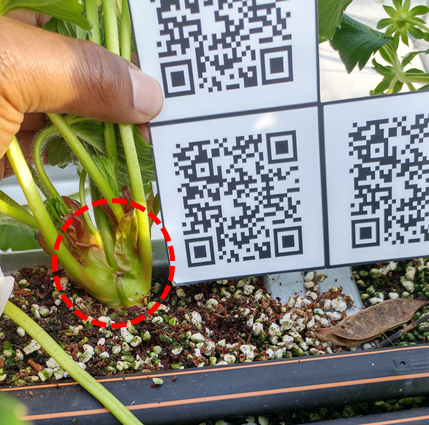** | To precisely measure the plant crown, hold the QR marker steadily with one hand, positioning it vertically and parallel to the crown on the side of the plant crown, near the soil where the leaf petioles are attached. Ensure the QR marker is aligned with the thickest part of the crown, as indicated by the dotted circle in the image. Capture the image by holding the camera perpendicularly to the crown and ensure that the thickest part of the crown and the QR marker are both clearly visible in one photo as indicated in the image. |

1. **Flower size (flower area)**

| **Phenotypic parameter** | **Measurement method** |
| --- | --- |
| **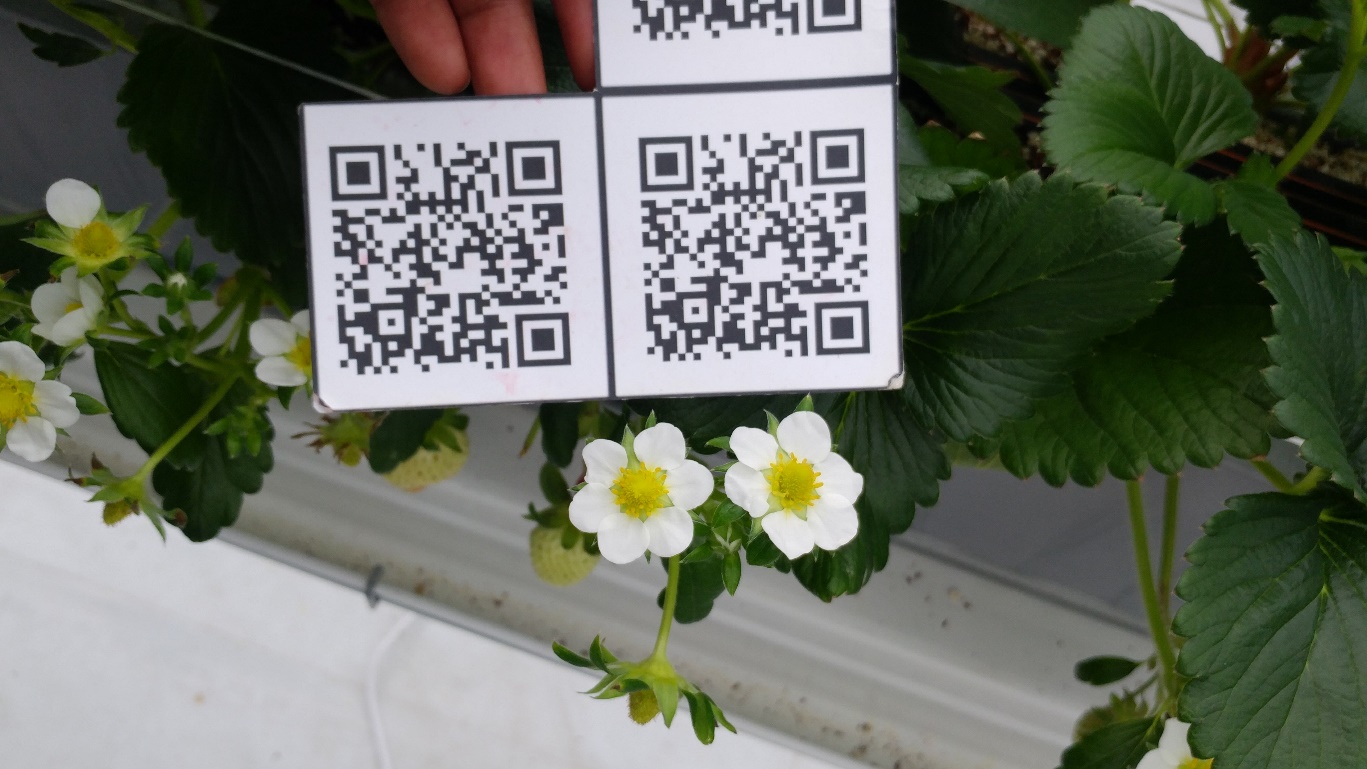** | Hold the QR marker steadily with one hand, positioning it beside the target flowers in the flower cluster. Ensure the QR marker and the target flower(s) are in the same plane and that the QR marker is relatively parallel to the target flowers. Hold the camera with your other hand and capture the image perpendicularly to the target flower(s), ensuring that the entire target flower(s) and QR marker are visible in the photo as indicated in the image. |

1. **Fruit size (fruit area)**

| **Phenotypic parameter** | **Measurement method** |
| --- | --- |
| **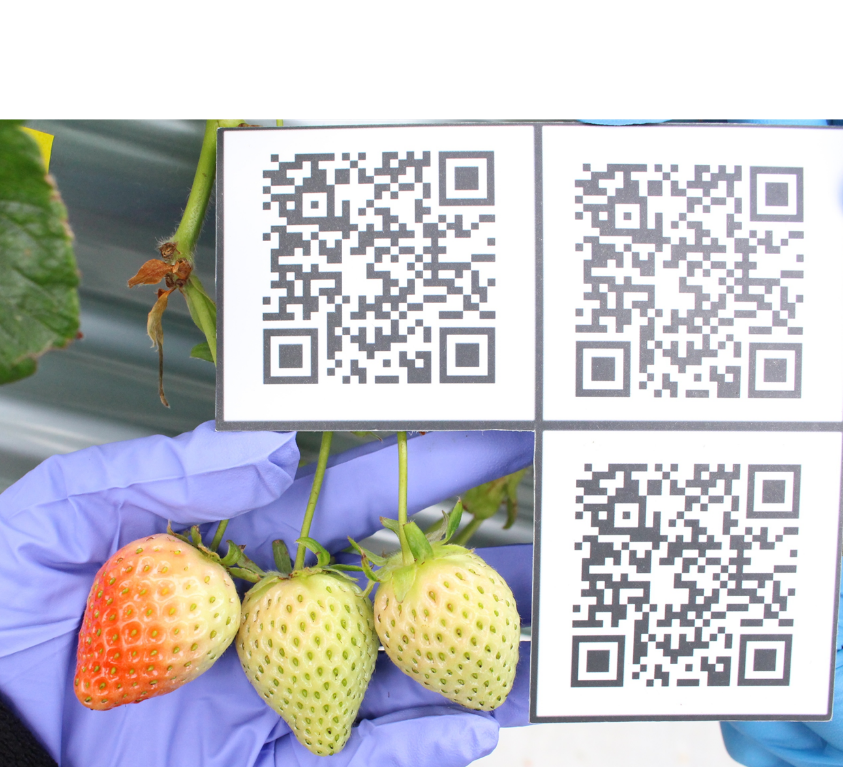** | Hold the QR marker steadily with one hand, positioning it beside the target fruit. Ensure the QR marker and the target fruit are in the same plane and that the QR marker is relatively parallel to the target fruit (s). Hold the camera with the other hand and capture the image perpendicularly to the target fruit (s), ensuring that the entire target fruit and QR marker are visible in the same photo as indicated in the image. |

1. **Visualizing and obtaining quantitative data**

Extracted data from images can be downloaded or displayed on the server for further analysis. Results are available in real-time, facilitating prompt decision-making.
